# Treatment of Local Paralysis by Phosphorus

**Published:** 1841-02

**Authors:** 


*Treatment of local Paralysis by Phosphorus.*—Several German writers very strongly recommend the use internally as well as externally, of phosphorus in cases of paralysis of the optic and auditory nerves.

In a case related by M. Dezeimeris in his late elaborate memoir on diseases and injuries of the frontal sinuses, where deafness and blindness on the left side supervened upon a severe injury of the head, he resorted to this treatment with success.

He prescribed as follows:

℞. Phosphori gr. j, solve in
Olei animalis æther ℥j,
Olei caryophyll. ℈j.

Dose.—Three drops, to be gradually increased to 20, to be taken on a piece of sugar night and morning. And

℞. Phosphori gr. ij.

Olei animalis æther ℥j.

Olei cajeputi ℥ss. Solve.

The eyelids to be well rubbed with this embrocation three or four times daily,

A blister also was applied upon the mastoid process and kept open.

In four weeks the patient had quite recovered his hearing and sight.—*Med. Chirurg. Rev.* Oct. 1840, from *L'Experience. Ibid.*

